# Retraction: The intra-nuclear SphK2-S1P axis facilitates M1-to-M2 shift of microglia via suppressing HDAC1-mediated KLF4 deacetylation

**DOI:** 10.3389/fimmu.2026.1809852

**Published:** 2026-02-20

**Authors:** Frontiers Editorial Office

The journal retracts the 04 June 2019 article cited above.

Following publication, concerns were raised about the integrity of images presented in the published figures, particularly duplicated images identified in **Figures 3F**, **4F**, and **5F**. Additional concerns were also noted regarding the integrity of the original western blot images provided during the subsequent investigation, which was carried out in compliance with Frontiers’ policies. As a result, the data and conclusions of the article have been deemed unreliable, and the article has been retracted.

The investigation followed Frontiers’ policies, and the retraction was approved by the Chief Executive Editor. The authors were informed about the retraction.

